# Role of recent and old riverine barriers in fine-scale population genetic structure of Geoffroy's tamarin (*Saguinus geoffroyi*) in the Panama Canal watershed

**DOI:** 10.1002/ece3.79

**Published:** 2012-02

**Authors:** Samuel L Díaz-Muñoz

**Affiliations:** 1 Museum of Vertebrate Zoology, University of CaliforniaBerkeley, California; 2 Department of Integrative Biology, University of CaliforniaBerkeley, California

**Keywords:** Geographic barrier, human modified, Panama Canal, population structure, Tamarin

## Abstract

The role of physical barriers in promoting population divergence and genetic structuring is well known. While it is well established that animals can show genetic structuring at small spatial scales, less well-resolved is how the timing of the appearance of barriers affects population structure. This study uses the Panama Canal watershed as a test of the effects of old and recent riverine barriers in creating population structure in *Saguinus geoffroyi*, a small cooperatively breeding Neotropical primate. Mitochondrial sequences and microsatellite genotypes from three sampling localities revealed genetic structure across the Chagres River and the Panama Canal, suggesting that both waterways act as barriers to gene flow. *F*-statistics and exact tests of population differentiation suggest population structure on either side of both riverine barriers. Genetic differentiation across the Canal, however, was less than observed across the Chagres. Accordingly, Bayesian clustering algorithms detected between two and three populations, with localities across the older Chagres River always assigned as distinct populations. While conclusions represent a preliminary assessment of genetic structure of *S. geoffroyi,* this study adds to the evidence indicating that riverine barriers create genetic structure across a wide variety of taxa in the Panama Canal watershed and highlights the potential of this study area for discerning modern from historical influences on observed patterns of population genetic structure.

## Introduction

The distribution of genetic variability across geography is affected by multiple biotic and abiotic factors (Loveless and Hamrick 1984; Avise 2004), including mode of reproduction, vagility, philopatry, and geography. The role of physical barriers in promoting population divergence and structure is well known (Avise and Felley 1979; Preziosi and Fairbairn 1992). While it is well established that animals can show genetic structuring at very small spatial scales (Selander 1970), the timing of the origin of those patterns has been more difficult to discern because they may be the combined result of contemporary processes (Zellmer and Knowles 2009) as well as longer term historical events (Bowen and Avise 1990). Thus, the timing and appearance of physical barriers and how quickly these affect genetic structure in populations remains a topic of interest (Matocq et al. 2000; Vandergast et al. 2007; Zellmer and Knowles 2009).

Landscape features that constitute barriers vary among species. For instance, differences in elevation contribute to population differentiation in two amphibians, the blotched tiger salamander (Spear et al. 2005) and the Columbia spotted frogs (Funk et al. 2005). Conversely, pacific jumping mice (*Zapus trinotatus*) readily bound large topographic barriers, with gene flow explained more appropriately by habitat connectivity (Vignieri 2005). Even relatively new and small barriers can affect the population structure of animals. Epps et al. (2005) showed that recent (∼40 yrs) anthropogenic barriers have caused a marked decline in genetic diversity, in a large vagile mammal, the desert bighorn sheep (*Ovis canadensis nelsoni*). Similarly, anthropogenic barriers that red grouse could theoretically cross in one flight acted as barriers to gene flow (Piertney et al. 1998).

Bodies of water can promote genetic differentiation in a variety of terrestrial species. Sea lochs explain most of the genetic differentiation among populations of red deer in the Scottish highlands (Pérez–Espona et al. 2008). Quéméré et al. (2010) found that the Manankolana River was the major barrier to gene flow for the golden crown sifaka (*Propithecus tattersalli*) in the Daraina region of Madagascar. Bodies of water may also be barriers for volant animals, as reported by (Meyer et al. 2009) for some bat species. Rivers can serve as barriers for amphibians, as reported for the alpine stream frog (*Scutiger* spp.) in the Hengduan Mountains of China (Li et al. 2009).

Although riverine barriers have been implicated as barriers to gene flow in a variety of species, less is known about how the timing of the appearance of these barriers affects genetic structure. The Panama Canal is one of the largest modifications of the hydrographic landscape undertaken by humans. Because the addition of this major riverine barrier is well documented historically and the Panama Canal is embedded in the center of a dynamic watershed with older riverine barriers, it presents an ideal opportunity to test the influence of the timing of physical barriers in population genetic differentiation. Before the construction of the Canal, the Chagres River flowed along the Atlantic slope of the Isthmus, while only small costal streams drained the Pacific slope (Meek and Hildebrand 1916, Fig. 2). Geologic studies indicate that the Panamanian Isthmus was formed by sometime in the Pliocene (Coates et al. 2004; Kirby et al. 2008), with little tectonic activity after the late Pliocene (Coates et al. 2004) indicating that by then the topographic composition of the Panama Canal watershed was probably similar to that observed today. Thus, while the Chagres River had been a major part of the central isthmian basin in paleontological time, the completion of the Panama Canal in 1914 created a novel riverine barrier, which is expected to have affected the movements and, hence, gene flow in a multitude of species.

**Figure 2:**
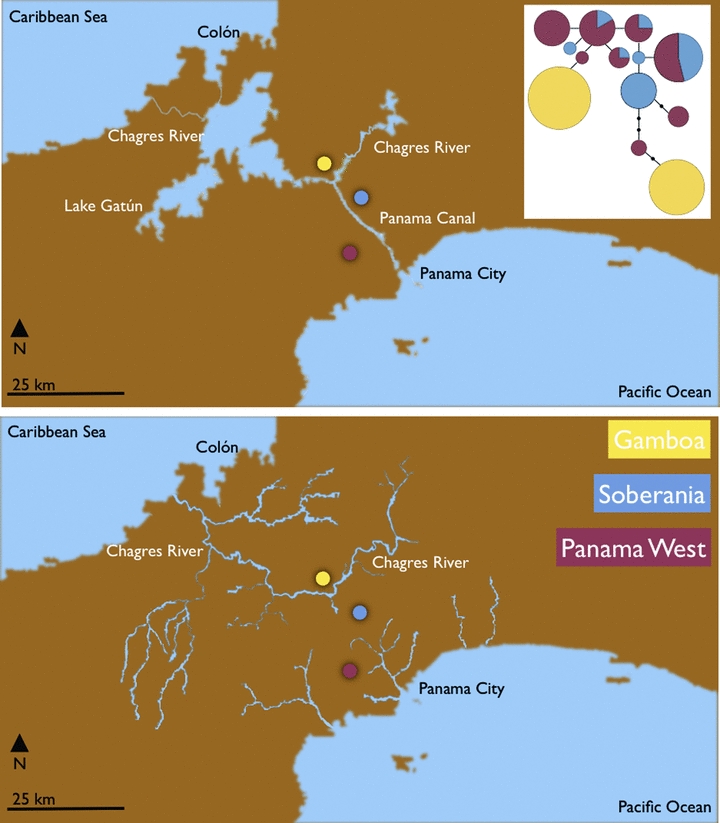
Sampling localities in the Panama Canal watershed. The top panel shows the current watershed, after the construction of the Canal. Inset shows the haplotype parsimony network generated by TCS, with haplotypes color coded to correspond with sampling localities and proportionally sized according to haplotype frequency. The bottom panel shows the watershed before the construction of the Canal.

**Figure 1:**
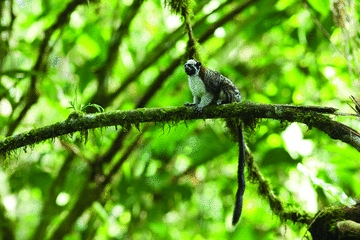
Geoffroy's tamarin in the Gamboa forest. Photo by Anand Varma.

This study uses the Panama Canal watershed to test the effects of an old and a recent riverine barrier in creating population structure in Geoffroy's tamarin *Saguinus geoffroyi*, a small Neotropical primate (Fig. 1). Tamarins represent an appropriate study species for this study for several reasons, first, the Chagres River (the old river) was in place as a major riverine barrier well before the estimated divergence time (0.7 Mya, Evans et al. 1998) of Geoffroy's tamarin from its sister species (*S. oedipus*). Second, previous work has shown that riverine barriers are important for structuring primate populations in general (Wallace 1852), and for the diversification of tamarins in particular. Hershkovitz (1977) originally proposed that subspecies of *S. fuscicollis* were delineated by rivers based on pelage color variation, a hypothesis later supported by analyses of craniofacial variation (Cheverud and Moore 1990). Peres et al. (1996) showed that cytochrome b haplotypes corresponded to phenotypically distinct morphs of subspecies of *S. fuscicollis* on opposite sides of the Rio Juruá in Amazonian Brazil. Furthermore, the authors showed that gene flow (and intergradation of color morphs) increased toward the narrow headwater streams of the river, as predicted by Wallace (1852). Third, among the primates inhabiting areas close to the Panama Canal (howler monkeys: *Alouatta palliata* and capuchin monkeys: *Cebus capuchinus*), tamarins may be most likely to exhibit rapid population differentiation in response to landscape changes. Tamarins exhibit high intragroup relatedness (Huck et al. 2005), suggesting a low dispersal rate and a greater likelihood of showing genetic structure. In contrast, howler monkeys inhabiting an isolated island in the Panama Canal watershed for > 90 years showed no genetic evidence of a population bottleneck (Milton et al. 2009), suggesting a near panmictic mating pattern.

To examine the role of rivers in creating population genetic structure at a small spatial scale, I sampled three populations distributed across two prominent riverine barriers—the Panama Canal and the Chagres River—to test whether the age of a physical barrier to gene flow has an effect on the level population genetic structure. I predict that there will be significant differentiation between populations separated by the Chagres, whereas differentiation across the Panama Canal will be more modest, owing to its novelty as a barrier: whereas the individuals included in this study represent approximately 20–40 generations since the construction of the Canal, the number of generations since the appearance of the Chagres is at least three orders magnitude higher. To test these predictions, I: (1) examine differences in genetic variability among sampling localities at mtDNA and microsatellite loci, (2) use *F*-statistics and analysis of molecular variance (AMOVA) at mtDNA and microsatellite loci to investigate population genetic structure between sampling localities, and (3) use Bayesian clustering algorithms to determine the number of likely populations.

## Materials and methods

### Study sites

I targeted three areas within the Panama Canal watershed (provinces of Panama and Colón, Republic of Panama) for sampling (Fig. 2): (a) The Soberanía field site located within the boundaries of Soberanía National Park, close to Camino de Plantación (9.076°, –79.659°). (b) The Gamboa field site, just outside the park boundaries, located in and around the rural town of Gamboa, Colón Province (9.118°, –79.698°). (c) The Panama West locality (8.957°, –79.668) west of Panama City across the Panama Canal. I selected the Soberanía and Gamboa field sites to provide a comparison across the Chagres River; these samples were collected as part of a separate study on cooperative breeding in *S. geoffroyi* (Díaz–Muñoz 2011). I selected the Panama West locality to capture the potential effect of the Panama Canal as a barrier. I collected samples from Soberanía National Park and Gamboa from field captures, whereas the samples from western Panama Province were obtained from museum skins collected by Dawson (1976) and housed at the Michigan State University Museum (Table 1). The use of museum samples imposed limitations on the study, specifically they represented a sample from a wider geographic area as compared to the other localities and represented a time period approximately 30 years earlier than field-collected samples (field samples: 2005–2009; museum samples: 1973–1974). However, I opted to use museum samples due to the fact that some areas directly across the Panama Canal from Soberanía have limited accessibility (due to deposits of unexploded ordnance from military activities) and tamarin populations at some localities in Western Panama have been extirpated due to growing urbanization. Utilizing museum resources allowed a broader sampling than would have been possible with field efforts and allowed looking at both of the riverine barriers of interest, albeit in a preliminary way.

**Table 1:** Michigan State University Museum specimens sampled for this study

Catalog no.	Sex	Year collected	Specific locality	Latitude	Longitude
MR.22872	M	1973	Cerro Cama	9.01667	–79.90000
MR.22874	M	1973	Vicinity of La Chorrera	8.88333	–79.78333
MR.22878	F	1973	15 km W of Balboa	8.95000	–79.70306
MR.22875	M	1973	15 km W of Balboa	8.95000	–79.70306
MR.22947	M	1973	6.5 km NW of Balboa	8.98197	–79.61785
MR.22885	M	1973	5 km NE of Arraijan	8.96598	–79.63393
MR.22891	M	1973	3.3 km NE of Arraijan	8.97110	–79.62878
MR.22889	F	1973	2.5 km NE of Arraijan	8.95000	–79.58635
MR.22998	F	1974	3 km W of Balboa	8.95000	–79.56816
MR.22989	M	1974	8.5 km W of Balboa	8.95000	–79.64850
MR.22935	F	1973	6 km SW of Balboa	8.92924	–79.61707
MR.22994	F	1974	8.5 km WSW of Balboa	8.92762	–79.58917
MR.22923	M	1973	3.5 km SW of Balboa	8.89885	–79.61811
MR.22963	F	1973	4 km ESE of Arraijan	8.91164	–79.60525
MR.22907	M	1973	9 km W of Balboa	8.99156	–79.60846
MR.22902	F	1973	9 km E of Arraijan	8.95000	–79.61363
MR.22949	F	1973	4 km E of Arraijan	8.93616	–79.61640
MR.22934	M	1973	8 km SW of Balboa	8.95865	–79.62900
MR.22895	F	1973	7 km E of Arraijan	8.96730	–79.60800
MR.22985	F	1973	5 km ENE of Arraijan	8.95000	–79.64396
MR.22980	F	1973	2.5 km ENE of Arraijan	8.92059	–79.63807
MR.22915	F	1973	6 km WSW of Balboa	8.95000	–79.59395

### Captures and sample collection

I captured tamarins from Gamboa and Soberanía using hand-activated live traps baited with bananas as described by Garber et al. (1993) or by blow-darting (BioJect, Blowguns Northwest, Richland, WA) with tranquilizer darts (Pnueu-Dart, Williamsport, PA). Individuals from the Soberanía population were only captured in traps and were not anesthetized. To prevent excessive stress, I limited handling time < 15 min and manipulations were constrained to marking and sampling hair. Soberanía individuals were released immediately at the capture site. I captured Gamboa adults using blow-darting; infants and juveniles were trapped because they were too small to safely dart. In both trapping and darting, I anesthetized Gamboa individuals using Ketamine (7.5 mg/kg) and Zoletil (3.75 mg/kg Vibrac SA, Carros Cedex, France). Gamboa animals were anesthetized to enable collection of morphological data for a separate study (Díaz–Muñoz 2011). I handled Gamboa individuals for 48 ± 14 min and placed them in a pet kennel for 3.67 ± 2.13 h until fully recovered. I measured respiration, heart rate, and internal body temperature of anesthetized individuals throughout handling procedures to monitor animal condition. To minimize potential injuries, I darted individuals at feeding stations that were eye-level above the ground. After darting, individuals were followed by two field assistants with a mesh net to catch anesthetized individuals that strayed from the feeding station. All capture and handling procedures were approved by the UC Berkeley Institutional Animal Care and Use Committee and followed the guidelines of the American Society of Mammalogists (Gannon and Sikes 2007).

Field collected samples included: (a) Hair samples plucked from the base of the tail and saved in coin envelopes and stored dry and (b) Ear tissue collected from the pinnae using surgical scissors and stored in RNA*later* and frozen at –20°C until extraction. Soberanía animals were represented by hair samples and Gamboa individuals were represented by hair and tissue samples. To verify the reliability of microsatellite genotypes from hair samples, I genotyped all Gamboa individuals using tissue samples to corroborate the results obtained from hair samples.

Museum skins were preserved as dry flat skins. I used surgical scissors to extract ca. 1 mm^2^ piece of tissue from the edge of the flat skin. I stored tissue samples in an empty microcentrifuge tube until extraction, which occurred within the week. A list of sampled individuals is included in Table 1.

### DNA extraction

I extracted Genomic DNA using Qiagen DNA Micro kits (Qiagen, Valencia, CA), according to manufacturer instructions for each sample type. I soaked museum tissue samples in 70% ethanol for 24 h prior to extraction. I quantified DNA yield using a NanoDrop Spectrophotometer (Thermo Fisher Scientific, Waltham, MA). I extracted DNA from hair and museum skin samples in a “clean” room dedicated to low-copy sample extractions. Both sampling and extraction negative controls were used to monitor for possible contamination at every step of the genetic workflow.

### Mitochondrial sequencing

Mitochondrial sequences are extensively used in population genetic studies (Avise 2004) owing to their uniparental inheritance and their relatively rapid sequence evolution, especially in the mitochondrial control region (Hoelzel et al. 1991). I amplified a 1080-bp fragment of the mitochondrial control region using primers designed to be genus-specific for *Saguinus* (Table 2; Cropp et al. 1999). To design primers, which would amplify short fragments suitable for skin and hair samples, I used control region sequences derived from tissue-extracted DNA from the Gamboa population. I amplified and sequenced the majority of the control region (ca. 1300 bp)—using “universal” primers MVZ 121/70 and MVZ 123/106 derived from other studies (Kocher et al. 1989; Palumbi 1996)—to design primers SGDL1-F, SGDL1-R, SGDL6-F, SGDL6-R, SGDL7-F, SGDL7-R using primer3 (Rozen and Skaletsky 2000), as implemented in Geneious 4.8.5 (Biomatters, Auckland, NZ). I performed PCR reactions on ABI 2720 (Applied Biosystems, Foster City, CA) or BioRad iCycler (Bio-Rad, Hercules, CA) thermocyclers using fluorescently labeled primers. Cycling conditions were 94°C for 4 min, 94°C for 1 min, 52°C for 1 min, 72°C for 75 sec, repeated 30 times; 72°C for 10 min. Polymerase chain reaction volume was 10 µl with 40 ng of genomic DNA, 1 µl of 10X PCR Buffer (Applied Biosystems), 2.5 mM MgCl2, 0.8 µl 10 mg/mL BSA, 0.4 mM of each DNTP, 3 pM of each primer, and 0.5 U of Taq polymerase (Invitrogen, Carlsbad, CA). I confirmed amplification via TBE (Tris-borate-EDTA) agarose gel electrophoresis and cleaned products of amplification using Exo-SAP IT (Affymetrix, Cleveland, OH). I fluorescently labeled PCR products utilizing ABI Big Dye 3.1 (Applied Biosystems) and sequenced amplicons in an ABI 3730 automated sequencer (Applied Biosystems). I aligned sequences using codoncode Aligner v3.5.6 (CodonCode, Dedham, MA) and geneious 4.8.5 (Biomatters). To ensure sequence accuracy, I obtained, aligned, and manually edited sequences from both strands (derived from each forward and reverse primer) belonging to the same individual. I used the consensus sequence of each individual for further analyses. I deposited consensus sequences in GenBank (JN849580-JN849633).

**Table 2:** Mitochondrial DNA primers used in study

Primer Name	5′-3′ sequence	Reference
SCJ5	TTGGTTATGTAATTAGTGC	Cropp et al. 1999
SCJ1	GAGCGAGAATACTAGTAGAAG	Cropp et al. 1999
464	TGAATTGGAGGACAACCAGT	Cropp et al. 1999
SCJ4	GCACTAATTACATAACCAA	Cropp et al. 1999
282	AAGGCTAGGACCAAACCT	Cropp et al. 1999
SCJ2	ACCCTTCAGAGAATAAACTTA	Cropp et al. 1999
SGDL1-F	GCACACGACTACCAAGCAAGATTATGA	This study
SGDL1-R	GGGTGGGGTGGGGACCAAGA	This study
SGDL6-F	TCATCAGCATTGCCGGGAGGC	This study
SGDL6-R	TGGTAGGCTAGGGGTATGTGGGG	This study
SGDL7-F	ACCCAAAAATCACCACCCTAAGCG	This study
SGDL7-R	TGGGGTTGTGACTGCCCATCT	This study

### Microsatellite genotyping

In order to maximize the chances of detecting population structure across the Panama Canal, I genotyped microsatellite loci. Due to their higher mutation rates (Jarne and Lagoda 1996), microsatellites provide information about genetic structure over shorter time scales than mtDNA, and provide a multilocus perspective on population genetic structure. I amplified seven polymorphic microsatellite loci (Table 3) from previously published studies (Escobar-Paramo 2000; Bohle and Zischler 2002) on ABI 2720 (Applied Biosystems) or BioRad iCycler (Bio-Rad) thermocyclers using fluorescently labeled primers. Cycling conditions followed the mitochondrial protocol with the following modifications: 35 cycles of amplification and locus-specific annealing temperature (see Ta, Table 3). I genotyped samples in an ABI 3730 automated sequencer (ABI, Foster City, CA): 1 µl of PCR product was added to 8.8 µl of formamide with 0.2 µl of GeneScan 500-LIZ size standard (ABI). I scored genotypes manually using genemapper 4.0 (ABI). To ensure robustness of genetic data, I typed homozygote genotypes from the museum skin samples and field collected hair samples from at least two independent PCR reactions. I genotyped a subset of samples from the Gamboa population from both hair and tissue samples. Additionally, a subset of samples was genotyped de novo from independent extractions. I deposited microsatellite genotypes in the Dryad data repository (doi:10.5061/dryad.mg87590q).

**Table 3:** Per-population microsatellite characteristics. Population means ± SD for each are presented

Population	Locus	N	Ta (°C)	Na	Ar	Par	Ho	He	UHe
Gamboa	Mean	19		2.86 ± 1.21	2.35 ± 0.57	0.19 ± 0.16	0.55 ± 0.21	0.50 ± 0.13	0.52 ± 0.14
	SB7	19	54	5	3.0636	0.3341	0.421	0.652	0.670
	SB8	19	54	2	1.8387	0.2213	0.421	0.332	0.341
	SB19	19	54	2	1.9801	0.0003	0.579	0.478	0.491
	SB38	19	54	2	1.8763	0.0959	0.474	0.361	0.371
	Ceb10	19	52	4	3.1310	0.2419	0.947	0.680	0.698
	Ceb11	19	52	3	2.5717	0.4081	0.684	0.532	0.546
	Ceb128	19	52	2	1.9913	0.0080	0.316	0.499	0.512
Panama W.	Mean	22		4.29 ± 1.80	2.85 ± 0.74	0.51 ± 0.55	0.54 ± 0.21	0.60 ± 0.14	0.61 ± 0.14
	SB7	22	54	5	3.2074	0.5249	0.455	0.658	0.673
	SB8	22	54	7	3.3217	1.3316	0.591	0.684	0.700
	SB19	21	54	2	1.9914	0.0012	0.524	0.500	0.512
	SB38	22	54	6	4.0426	1.2063	0.955	0.785	0.803
	Ceb10	22	52	3	2.7673	0.0774	0.500	0.624	0.638
	Ceb11	22	52	4	2.6181	0.2420	0.455	0.567	0.580
	Ceb128	22	52	3	1.9836	0.1592	0.273	0.361	0.369
Soberanía	Mean	18		4.14 ± 2.34	2.87 ± 0.99	0.48 ± 0.58	0.61 ± 0.19	0.57 ± 0.16	0.58 ± 0.17
	SB7	17	54	8	4.2180	1.4753	0.882	0.782	0.806
	SB8	16	54	6	3.6544	1.0413	0.750	0.689	0.712
	SB19	17	54	2	1.9356	0.0001	0.588	0.415	0.428
	SB38	18	54	5	3.7931	0.4310	0.722	0.748	0.770
	Ceb10	18	52	4	2.5491	0.4001	0.444	0.451	0.463
	Ceb11	18	52	2	1.9584	0.0142	0.333	0.444	0.457
	Ceb128	17	52	2	1.9544	0.0004	0.529	0.438	0.451

N = number of individuals typed; Ta = annealing temperature for reaction; Na = number of alleles loci used in this study; Ar = allelic richness. Par = private allelic richness; Ho = observed heterozygosity; He = expected heterozygosity; UHe = unbiased heterozygosity.

I checked loci for evidence of null alleles and genotyping errors using the program Microchecker 2.2 (van Oosterhout et al. 2004). I tested microsatellite loci for deviations from Hardy–Weinberg and linkage disequilibrium using Fstat 2.93 (Goudet 1995).

### Genetic diversity

I used the program dnasp 5.1 (Librado and Rozas 2009) to calculate nucleotide (π) and haplotypic (*h*) diversity for mitochondrial sequence data. I calculated number of alleles, expected and observed heterozygosities for microsatellite loci using arlequin 3.1 and genalex 6.1 (Peakall and Smouse 2006). I calculated allelic richness and private allelic richness using the rarefaction method (to control for sample size differences) in hp-rare 1.1 (Kalinowski 2005). I tested for differences between populations in microsatellite genetic diversity statistics using analysis of variance (ANOVA). Statistical tests are two-tailed and I report means with their standard deviations (mean ± SD), unless otherwise noted.

### Population genetic structure

I examined mitochondrial haplotype relationships using a parsimony network as calculated by Tcs 1.22 (Clement et al. 2000), with a 95% connection limit. I conducted an exact test of population differentiation based on mitochondrial haplotype frequencies (Raymond and Rousset 1995) in arlequin 3.1 (Excoffier et al. 2005). I calculated pairwise *F*-statistics in arlequin to investigate population genetic structure via mtDNA and microsatellite data. I tested for differences in Fst values between populations using 10,100 permutations. I used analysis of molecular variance, AMOVA, (Excoffier et al. 1992) to examine the amount of genetic variance explained by within- and among-population (i.e., sampling locality) variation.

### Population assignment

To determine the probable number of populations represented by the dataset, I used three different Bayesian clustering methods, due to previously reported variability in performance of these methods (Waples and Gaggiotti 2006; Rowe and Beebee 2007) and the somewhat different algorithms implemented and information used in each program. I ran Structure (Pritchard et al. 2000) that was run with no previous population information (USEPOPFLAG = 0), using an admixture model and assuming allele frequencies were correlated. I conducted four replicate runs at *k* = 1–5 using a burin of 10^6^ and a run length of 10^9^. I selected the optimal *k* value using the highest Pr(X|k) values (Pritchard et al. 2000). I conducted an assignment test in Geneclass2 (Piry et al. 2004) to determine the probability that each individual was assigned to its own “population,” in this case representing the sampling locality. I also ran the analysis with an alternate population definition collapsing the Soberanía and Panama West individuals into one population. Individuals were assigned to a population using the criterion of Rannala and Mountain (1997) and the probability of these assignments was calculated using a Monte-Carlo resampling technique (Piry et al. 2004), based on 10,000 simulated individuals. The Type I error was set at 0.01. I used Geneland as a third method of population clustering because it incorporates spatial data in order to identify genetic discontinuities in a spatially explicit fashion, which is relevant to the question at hand. First, I determined the most probable number of *k* populations using the Markov chain Monte Carlo (MCMC) over 5 × 10^5^ iterations (Coulon et al. 2004), using the uncorrelated model. I conducted five replicates of this process to ensure consistency of results (Rowe and Beebee 2007). I generated spatial maps of the posterior probability of population membership by using the posterior probability of population membership obtained from the MCMC simulation. I overlaid the course of the Chagres River and the Panama Canal (extracted from a satellite photo) was overlaid on the population map, in order to investigate the coincidence of geographic barriers with the population limits calculated by Geneland.

## Results

Genetic samples were collected from a total of 59 *S. geoffroyi* across the Panama Canal watershed. The number of individuals from which microsatellite (and mitochondrial) data were collected in each population were: Gamboa: 19 (17), Panama West: 22 (21), and Soberanía: 18 (16).

### Microsatellite genotyping

Microsatellite loci showed no evidence of null alleles or genotyping errors. Loci did not show evidence of departure from Hardy–Weinberg equilibrium or linkage disequilibrium in any of the populations after correction for multiple tests at the 0.05 nominal level. Genotyping of hair samples and ear tissue samples from Gamboa yielded identical genotypes at all loci, suggesting that genotypes from hair samples were reliable.

### Genetic diversity

Analysis of mitochondrial sequences revealed 12 variable sites and 13 haplotypes. As expected for a larger geographic sample, the Panama West population had the largest number of haplotypes, but it did not have the greatest nucleotide diversity, as did Gamboa (Table 4). Measures of allelic diversity at microsatellite loci (Table 3) were not significantly different between sampling localities (ANOVA's: Observed heterozygosity *P* = 0.8008, unbiased heterozygosity *P* = 0.5118, allelic richness *P* = 0.3991, rarefaction-calculated private allelic richness *P* = 0.3882). Gamboa appeared to have the lowest allelic diversity across measures, but these differences were not statistically significant. The lack of difference in microsatellite diversity included the Panama West locality, despite the differences in the nature of the sampling regime (larger geographic extent, different time frame).

**Table 4:** Diversity statistics for mitochondrial sequence data. Standard deviation is for sampling and stochastic processes

Population	N	Number of Haplotype	π Nucleotide diversity	*h* haplotypes diversity
Panama West	21	8	0.00289 ± 0.00047	0.886 ± 0.036
Gamboa	17	2	0.00392 ± 0.00033	0.529 ± 0.045
Soberanía	16	7	0.00171 ± 0.00026	0.792 ± 0.076

**N** = number of individuals sequenced in each population.

### Population structure

The haplotype network (Fig. 2) revealed sharing of haplotypes between the Panama West and Soberanía sites. The number of haplotypes observed in the Panama West locality was larger than at the other localities. The Gamboa locality was composed of two haplotypes, which were not shared with the other populations. An exact test based on haplotype frequencies suggested strong evidence for differentiation of three populations (*P* < 0.0001; 30,000 Markov steps).

Permutation analyses of fst values yielded significantly different (*P* < 0.001) values between all pairwise comparisons of sampling localities (Table 5). All fst values were statistically significantly different from zero. fst values calculated from mitochondrial sequence data were in general over two times larger than those calculated from microsatellite data. In each case, fst values calculated from localities across the Chagres River were around two times more divergent relative to comparisons across the Panama Canal, suggesting greater differentiation across the older riverine barrier.

**Table 5:** Pairwise comparisons for *F*-statistics using mtDNA and microsatellite data as calculated by ARLEQUIN. Microsatellite data are above the diagonal and shaded. Statistically significant differences at ^*^P < 0.001 and ^*^^*^P < 0.0001, respectively. *P* values calculated based on 10,100 permutations

Population	Panama West	Gamboa	Soberanía
Panama West		0.13616^*^	0.06428^*^
Gamboa	0.28532^*^		0.13247^*^
Soberanía	0.12061^*^^*^	0.34123^*^^*^	

AMOVA for mitochondrial data attributed 27.85% of variance to among-group (sampling locality) variation, compared to 11.11% for microsatellite data (Table 6). The fixation index and among-population variance components calculated by both AMOVA's differed significantly from random expectation (*P* < 0.0001).

**Table 6:** Analysis of molecular variance for microsatellites and mitochondrial (in parentheses) data. Based on 10,100 permutations (*P* < 0.0001)

Source of variation	df	Sum of squares	Variance components	Percentage of variation
Among populations	2 (2)	22.95 (24.47)	0.24 (0.60)	11.11 (27.85)
Within populations	115 (51)	223.70 (79.00)	1.95 (1.55)	88.89 (72.15)
Total	117 (53)	246.703 (103.46)	2.19 (2.15)	
Fixation index	fst: 0.11 (0.28)			

### Population assignment

**Figure 4:**
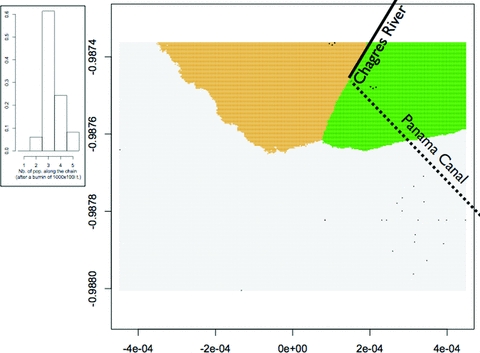
Map of population membership as calculated by geneland. Pixels are colored according to the modal posterior probability of population membership. The approximate location of Panama Canal and the Chagres River (drawn using georeferenced satellite images) are indicated in black. The inset shows the density of the estimate of *k* (number of populations) along the Markov chain.

The three Bayesian methods yielded different estimates for most probable number of populations. Structure detected *k* = 2 as the most likely number of populations, with the Gamboa locality distinct from the combined Panama West and Soberanía localities. The assignment plot is depicted in Figure 2. Geneclass2 analysis correctly assigned 78% of individuals to their sampling localities when three populations were assumed. When two populations were assumed, assignment success increased to 88.1% of individuals as did the quality index (*k* = 3: 62.06%*k* = 2: 80.77%), which represents the mean value of individual assignment scores (Piry et al. 2004). Both Structure and geneclass2 recovered localities across the Chagres as distinct populations, but did not always recover two distinct populations when comparing localities across the Panama Canal. Geneland on the other hand, clearly delineated three populations with minimal variance in the posterior probabilities of population estimation over multiple runs. The location of the riverine barriers under study were largely consistent with the population limits delineated by Geneland (Fig. 4).

## Discussion

The results suggest that both the Chagres River and the Panama Canal have contributed to population genetic structure in *S. geoffroyi* inhabiting the Panama Canal watershed. Although the sampling regime is limited, the results provide good, albeit preliminary, evidence of differentiation across two riverine barriers. Analyses of *F*-statistics, haplotypic data, and output from Bayesian assignment algorithms are collectively consistent with the Chagres River playing a role in relatively strong population differentiation, especially considering the small geographic distances between sampling localities (∼6 km). All analyses except Structure and Geneclass2, indicate that there is detectable population differentiation among sampling localities across the Panama Canal. As expected, the level of differentiation was smaller across the Panama Canal than across the Chagres River.

There were possible limitations imposed by the sampling regime for interpreting differentiation of sampling localities across the Panama Canal. In particular, the wider geographic sampling at the Panama West locality may cause additional allelic and haplotypic variation to be sampled. Analyses of microsatellite data suggest that this is not the case. Although the number of haplotypes is larger in the Panama West locality, it was Gamboa that had the greatest nucleotide diversity. However, because of these differences in sampling regime, the results of this study should be interpreted as preliminary evidence. More generally, the limited number of sampling localities also underscores the need for caution absent broader geographic sampling. Previous studies investigating the role of riverine barriers have found discrepant results when sampling at different localities along riverine barriers (e.g., Patton et al. 1994). Future studies incorporating several populations across a larger area in the Canal watershed will allow quantitative tests of the role of riverine barriers in creating population structure in *S. geoffroyi*.

### Differentiation discrepancies according to marker type

The degree of differentiation among sampling localities inferred using mitochondrial data was larger that that calculated with microsatellite genotypes. This was true across both the Chagres River and the Panama Canal. The AMOVA conducted on both genetic datasets indicated that a greater proportion (almost three times) of among-group variance was explained by mitochondrial sequence differences, as expected for a uniparentally inherited marker.

### Number of distinct populations using Bayesian clustering

**Figure 3:**
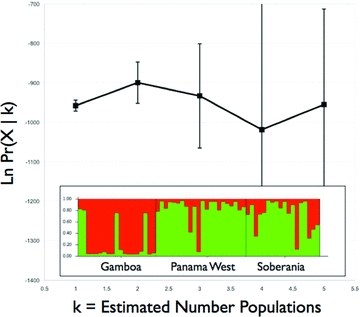
Posterior probability of each *k* estimate of structure. Error bars are the variance of the posterior probability estimate. Inset is the bar plot for the most likely number of populations (*k* = 2), which shows the fractional assignment probability to each individual to the clusters inferred by structure.

Although the results from Structure and Geneclass2 suggest two populations in the dataset, Bayesian algorithms have been reported to perform poorly at detecting populations with low differentiation (Waples and Gaggiotti 2006). Moreover, the creators of Structure caution that the large parameter space complicates the selection of *k* (Pritchard et al. 2000). This situation seems applicable to the current study as evidenced by the overlapping variances of Pr(X|k) for two and three populations (Fig. 3). On the other hand, Geneland consistently identified three populations and the geographic projection of population membership probabilities roughly coincided with the approximate location of both putative barriers under study. These results underscore the variability of *k* estimates from different population clustering algorithms and suggest that future researchers should use multiple methods (Rowe and Beebee 2007), and evaluate results in light of the biological significance to the study species (Pritchard et al. 2000).

### Riverine barriers in the Panama Canal watershed

The Chagres River has been associated with genetic structure in at least one other species. (Lampert at al. 2003) showed that the Chagres River formed a barrier to dispersal of túngara frogs as indicated by isolation by distance patterns calculated using microsatellite markers. Evidence that the Panama Canal has affected gene flow in a multitude of species is more abundant. (Meyer et al. 2009) showed that bat populations inhabiting the islands created upon the flooding of Gatún Lake had lower genetic diversity and higher genetic differentiation than mainland populations, according to their dispersal abilities. Studies of freshwater fish suggest that distinct species assemblages existed on either side of the Cordillera Central on the Atlantic and Pacific Slopes of the Isthmus (Meek and Hildebrand 1916; Smith et al. 2004) and now exist in the same communities as a consequence of the aquatic connection provided by the Canal (Smith et al. 2004). Thus, the Panama Canal has affected population structure in a variety of taxa, increasing gene flow in aquatic species and restricting it in some terrestrial species.

Demographic evidence also supports the idea of reduced gene flow in terrestrial species as a consequence of the creation of the Panama Canal. Intensive studies on Barro Colorado Island (BCI) have shown that multiple bird species have become locally extinct, most likely as a cause of the limited dispersal across the Canal (Robinson 1999). There is also demographic evidence that the canal had significant effects on *S. geoffroyi* populations: in BCI, the tamarin population has seen decline, as observational (Enders 1939; Eisenberg & Thorington 1973) and census data (Wright et al. 2000) suggests. While habitat conversion (from secondary to primary forest) has been suggested as a cause of the decline of tamarins on BCI, the results of this study and those cited above suggest that the absence of dispersal and gene flow could have played a part in this demographic change.

### Conservation implications

The lower differentiation across the Panama Canal suggested by this study points to only modest structure. However, in the absence of migrants these populations may diverge in the future, as has happened more clearly across the Chagres. In fact, divergence may be hastened across the Panama Canal, due to decreasing habitat availability west of the Panama Canal. In contrast, the Chagres headwaters are < 25 km from both Soberanía and Gamboa populations and well forested. This may ensure that over time gene flow will persist across, that is around, this barrier, which will likely not be the case for the Panama Canal. This study adds to the growing body of literature on the effects of recent anthropogenic barriers on population structure and genetic diversity (Piertney et al. 1998; Smith et al. 2004; Epps et al. 2005). The results of this study highlight the utility of the Panama Canal watershed as an ideal testing ground for questions of population structure. Moreover, the proximity of natural protected areas to two rapidly growing population centers (Rompre et al. 2008) provide challenges for species conservation, but ample opportunities for conservation-oriented biological research on a number of tropical species. It is hoped that the current study will stimulate such research.

## References

[b1] Avise JC (2004). Molecular markers, natural history, and evolution.

[b2] Avise JC, Felley J (1979). Population-structure of freshwater-fishes 1. genetic-variation of bluegill (*Lepomis macrochirus*) populations in man-made reservoirs. Evolution.

[b3] Bohle UR, Zischler H (2002). Polymorphic microsatellite loci for the mustached tamarin (*Saguinus mystax*) and their cross-species amplification in other New World monkeys. Mol. Ecol. Notes.

[b4] Bowen B, Avise JC (1990). Genetic-structure of Atlantic and Gulf of Mexico populations of sea bass, menhaden, and sturgeon – influence of zoogeographic factors and life-history patterns. Mar. Biol.

[b5] Cheverud JM, Moore AJ (1990). Subspecific Morphological Variation In The Saddle-Back Tamarin (*Saguinus-fuscicollis*. Am. J. Primatol.

[b6] Clement M, Posada D, Crandall KA (2000). TCS: a computer program to estimate gene genealogies. Mol. Ecol.

[b7] Coates AG, Collins LS, Aubry MP, Berggren WA (2004). The geology of the Darien, Panama, and the late Miocene-Pliocene collision of the Panama arc with northwestern South America. Geol. Soc. Am. Bull.

[b8] Coulon A, Cosson JF, Angibault JM, Cargnelutti B, Galan M, Morellet N, Petit E, Aulagnier S, Hewison AJM (2004). Landscape connectivity influences gene flow in a roe deer population inhabiting a fragmented landscape: an individual-based approach. Mol. Ecol.

[b9] Cropp SJ, Larson A, Cheverud JM (1999). Historical biogeography of tamarins, genus *Saguinus*: the molecular phylogenetic evidence. Am. J. Phys. Anthropol.

[b10] Dawson G (1976). Behavioral ecology of the Panamanian Tamarin, *Saguinus Oedipus* (Callitrichidae, Primates).

[b11] Díaz-Muñoz SL (2011). Paternity and relatedness in a polyandrous nonhuman primate: testing adaptive hypotheses of male reproductive cooperation. Anim. Behav.

[b12] Eisenberg JF, Thorington RW (1973). A preliminary analysis of a neotropical mammal fauna. Biotropica.

[b13] Enders RK (1939). Changes observed in the mammal fauna of Barro Colorado Island. Ecology.

[b14] Epps CW, Palsboll PJ, Wehausen JD, Roderick GK, Ramey RR, McCullough DR (2005). Highways block gene flow and cause a rapid decline in genetic diversity of desert bighorn sheep. Ecol. Lett.

[b15] Escobar-Paramo P (2000). Microsatellite primers for the wild brown capuchin monkey *Cebus apella*. Mol. Ecol.

[b16] Evans DT, Piekarczyk MS, Cadavid L, Hinshaw VS, Watkins DI (1998). Two different primate species express an identical functional mhc class i allele. Immunogenetics.

[b17] Excoffier L, Smouse P, Quattro J (1992). Analysis of molecular variance inferred from metric distances among DNA haplotypes – application to human mitochondrial-DNA restriction data. Genetics.

[b18] Excoffier L, Laval G, Schneider S (2005). Arlequin (version 3.0): an integrated software package for population genetics data analysis. Evol. Bioinform.

[b19] Funk WC, Blouin MS, Corn PS, Maxell BA, Pilliod DS, Amish S, Allendorf FW (2005). Population structure of Columbia spotted frogs (*Rana luteiventris*) is strongly affected by the landscape. Mol. Ecol.

[b20] Gannon W, Sikes R (2007). Guidelines of the American Society of Mammalogists for the use of wild mammals in research. J. Mammal.

[b21] Garber PA, Encarnacion F, Moya L, Pruetz JD (1993). Demographic and reproductive patterns in moustached tamarin monkeys (*Saguinus mystax*) – implications for reconstructing platyrrhine mating systems. Am. J. Primatol.

[b22] Goudet J (1995). FSTAT (Version 1.2): a computer program to calculate F-statistics. J. Hered.

[b23] Hershkovitz P (1977). Living new world monkeys.

[b24] Hoelzel AJ, Hancock J, Dover G (1991). Evolution of the cetacean mitochondrial d-loop region. Mol. Biol. Evol.

[b25] Huck M, Löttker P, Böhle UR, Heymann EW (2005). Paternity and kinship patterns in polyandrous moustached tamarins (*Saguinus mystax*. Am. J. Phys. Anthropol.

[b26] Jarne P, Lagoda PJL (1996). Microsatellites, from molecules to populations and back. Trends Ecol. Evol.

[b27] Kalinowski ST (2005). HP-RARE 1.0: a computer program for performing rarefaction on measures of allelic richness. Mol. Ecol. Notes.

[b28] Kirby MX, Jones DS, MacFadden BJ (2008). Lower miocene stratigraphy along the Panama Canal and its bearing on the Central American peninsula. Plos One.

[b29] Kocher TW, Thomas W, Meyer A, Edwards S, Paabo A, Villablanca F, Wilson A (1989). Dynamics of mitochondrial-DNA evolution in animals – amplification and sequencing with conserved primers. Proc. Natl. Acad. Sci. U.S.A.

[b30] Lampert KP, Rand AS, Mueller UG, Ryan MJ (2003). Fine-scale genetic pattern and evidence for sex-biased dispersal in the tungara frog, *Physalaemus pustulosus*. Mol. Ecol.

[b31] Li R, Chen W, Tu L, Fu J (2009). Rivers as barriers for high elevation amphibians: a phylogeographic analysis of the alpine stream frog of the Hengduan mountains. J. Zool.

[b32] Librado P, Rozas J (2009). DnaSP V5: a software for comprehensive analysis of DNA polymorphism data. Bioinformatics.

[b33] Loveless M, Hamrick J (1984). Ecological determinants of genetic-structure in plant-populations. Ann. Rev. Ecol. Syst.

[b34] Matocq MD, Patton JL, da Silva M (2000). Population genetic structure of two ecologically distinct Amazonian spiny rats: separating history and current ecology. Evolution.

[b35] Meek SE, Hildebrand SF (1916). The fishes of the fresh waters of Panama.

[b36] Meyer CFJ, Kalko EKV, Kerth G (2009). Small-scale fragmentation effects on local genetic diversity in two phyllostomid bats with different dispersal abilities in Panama. Biotropica.

[b37] Milton K, Lozier J, Lacey E (2009). Genetic structure of an isolated population of mantled howler monkeys (*Alouatta palliata*) on Barro Colorado Island, Panama. Conserv. Genet.

[b38] Palumbi SR, Hillis DM, Moritz C, Mable BK (1996). Nucleic Acids II: the polymerase chain reaction. Molecular systematics.

[b39] Patton JL, da Silva MNF, Malcolm JR (1994). Gene genealogy and differentiation among arboreal spiny rats (Rodentia, Echimyidae) of the Amazon Basin – a test of the riverine barrier hypothesis. Evolution.

[b40] Peakall R, Smouse P (2006). GENALEX 6: genetic analysis in excel. Population genetic software for teaching and research. Mol. Ecol. Notes.

[b41] Peres C, Patton JL, da Silva MNF (1996). Riverine barriers and gene flow in Amazonian saddle-back tamarins. Folia Primatol.

[b42] Pérez-Espona S, Perez-Barberia FJ, McLeod JE, Jiggins CD, Gordon IJ, Pemberton JM (2008). Landscape features affect gene flow of Scottish highland red deer (*Cervus elaphus*. Mol. Ecol.

[b43] Piertney SB, MacColl ADC, Bacon PJ, Dallas JF (1998). Local genetic structure in red grouse (*Lagopus lagopus scoticus*): evidence from microsatellite DNA markers. Mol. Ecol.

[b44] Piry S, Alapetite A, Cornuet JM, Paetkau D, Baudouin L, Estoup A (2004). GENECLASS2: a software for genetic assignment and first-generation migrant detection. J. Hered.

[b45] Preziosi R, Fairbairn D (1992). Genetic population-structure and levels of gene flow in the stream dwelling waterstrider, *Aquarius* ( = Gerris) *remigis* (Hemiptera, Gerridae). Evolution.

[b46] Pritchard JK, Stephens M, Donnelly P (2000). Inference of population structure using multilocus genotype data. Genetics.

[b47] Quéméré E, Crouau-Roy B, Rabarivola C, Louis EE, Chikhi L (2010). Landscape genetics of an endangered lemur (*Propithecus tattersalli*) within its entire fragmented range. Mol. Ecol.

[b48] Rannala B, Mountain JL (1997). Detecting immigration by using multilocus genotypes. P. Natl. Acad. Sci. U.S.A.

[b49] Raymond M, Rousset F (1995). An exact test for population differentiation. Evolution.

[b50] Robinson WD (1999). Long-term changes in the avifauna of Barro Colorado Island, Panama, a tropical forest isolate. Conservation Biology.

[b51] Rompre G, Robinson WD, Desrochers A (2008). Causes of habitat loss in a neotropical landscape: the Panama Canal corridor. Landscape Urban Plan.

[b52] Rowe G, Beebee TJC (2007). Defining population boundaries: use of three Bayesian approaches with microsatellite data from British natterjack toads (*Bufo calamita*. Mol. Ecol.

[b53] Rozen S, Skaletsky H, Krawetz S, Misener S (2000). Primer3 on the WWW for general users and for biologist programmers. Bioinformatics methods and protocols: methods in molecular biology.

[b54] Selander R (1970). Behavior and genetic variation in natural populations. Am. Zool.

[b55] Smith SA, Bell G, Bermingham E (2004). Cross-Cordillera exchange mediated by the Panama Canal increased the species richness of local freshwater fish assemblages. Proc. R. Soc. Lond. B.

[b56] Spear SF, Peterson CR, Matocq MD, Storfer A (2005). Landscape genetics of the blotched tiger salamander (*Ambystoma tigrinum melanostictum*. Mol. Ecol.

[b57] Vandergast AG, Bohonak AJ, Weissman DB, Fisher RN (2007). Understanding the genetic effects of recent habitat fragmentation in the context of evolutionary history: phylogeography and landscape genetics of a southern California endemic Jerusalem cricket (Orthoptera: Stenopelmatidae: *Stenopelmatus*. Mol. Ecol.

[b58] van Oosterhout C, Hutchinson WF, Wills DPM, Shipley P (2004). MICRO-CHECKER: software for identifying and correcting genotyping errors in microsatellite data. Mol. Ecol. Notes.

[b59] Vignieri SN (2005). Streams over mountains: influence of riparian connectivity on gene flow in the Pacific jumping mouse (*Zapus trinotatus*. Mol. Ecol.

[b60] Wallace AR (1852). On the monkeys of the Amazon. Proc. Zool. Soc. Lond.

[b61] Waples R, Gaggiotti O (2006). What is a population? An empirical evaluation of some genetic methods for identifying the number of gene pools and their degree of connectivity. Mol. Ecol.

[b62] Wright SJ, Zeballos H, Dominguez I, Gallardo MM, Moreno MC, Ibanez R (2000). Poachers alter mammal abundance, seed dispersal and seed predation in a neotropical forest. Conservation Biology.

[b63] Zellmer AJ, Knowles LL (2009). Disentangling the effects of historic vs. contemporary landscape structure on population genetic divergence. Mol. Ecol.

